# Durability Enhancement Effect of Silica Fume on the Bond Behavior of Concrete–PCM Composites under Environmental Conditions

**DOI:** 10.3390/polym15092061

**Published:** 2023-04-26

**Authors:** Mahmudul Hasan Mizan, Koji Matsumoto

**Affiliations:** 1 Graduate School of Engineering, Hokkaido University, Hokkaido 060-0808, Japan; 2 Department of Civil Engineering, Khulna University of Engineering & Technology, Khulna 9203, Bangladesh

**Keywords:** long-term performance, concrete–PCM interface, environmental exposure (elevated temperature and moisture content), interface strength, silica fume

## Abstract

The long-term performance of the concrete–polymer cement mortar (PCM) interface under environmental exposure is crucial to the safety of the PCM overlaying method as the environmental exposure of the repaired structures caused further degradation of the interface, leading to a significant reduction in intended service life. This study investigates the durability enhancement effect of silica fume of the concrete–PCM interface, considering an individual action of elevated temperature (e.g., 60 °C) [constant (short and moderate duration) and cyclic conditions] and moisture content [continuous immersion and wetting/drying (W/D) cycle]. Our previous research confirmed that the use of silica fume forms more C-S-H with strong binding force and enhances the interfacial bonding strength due to the denser microstructure at the interface, and it is expected to be utilized for durability purposes under the aforementioned exposure conditions. Under all elevated temperature exposure conditions, the reduction percentage of the interfacial performance corresponding to the respective reference specimens reduced significantly with the inclusion of silica fume with overlay material. The occurrence of interface fracture at lower load and a greater number of pure interface fracture modes observed in normal PCM specimens compared to modified PCM specimens indicates a positive influence of higher adhesion with better durability of modified PCM overlay with substrate concrete. Under both conditions of moisture content, significant reduction in interfacial strength was observed in normal PCM specimens. In all cases, the reducing ratio of interfacial strength was higher in normal PCM compared to modified PCM, indicating a positive influence of silica fume under moisture content. Furthermore, silica fume inclusion shifts the fracture mode from pure interfacial fracture to composite fracture mode, indicating a positive response of silica fume to improve the resistance of interface fracture under moisture content. Conclusively, the use of silica fume improves concrete–overlay layer adhesion and enhances the bonding durability under environmental exposure.

## 1. Introduction

The performance of mortar or concrete significantly improves with the use of functional polymer materials or admixtures, as observed in several past experimental studies [1,2,3,4,5,6]. Different nanomaterials such as zinc oxide, titanium dioxide, or industrial waste with fibers were also used as a partial replacement of cement to prepare concrete or mortar with long-term strength and durability [7,8]. Recently, PCM (polymer cement mortar) has become the dominant material in the construction industry and is used as a popular repairing material as it possesses very compatible properties with respect to workability, adhesion, frost resistance, durability, permeability, and rate of shrinkage with concrete [9,10,11,12,13,14,15]. The PCM overlaying method has broad application prospects to improve load resistance, reduce deformation, and alleviate stress in existing structures, such as dams, bridge decks, and highway pavement slabs [16,17,18]. The concrete–PCM interfacial bonding properties are considered a very important factor. Many experimental studies in the past [19,20,21] observed a weakest link at the concrete–PCM interface of the PCM overlaying method. In technical literature, many studies revealed that a supplementary cementitious material such as silica fume can be used as a filler, pozzolan, or admixture to improve the performance of mortar and concrete [22,23,24,25]. In the previous study [26,27,28,29,30], it has also been shown that silica fume modified PCM as a repair material significantly improves the concrete–PCM interfacial bond, thus offering a broad prospect for the application of the PCM overlaying method for the strengthening of deteriorated reinforced concrete (RC) structures.

In practice, the strengthened structures are put into immediate service based on achieving adequate substrate concrete–repair materials bond strength in a short duration. In many cases, the short-term strength gain of the strengthened members does not ensure long-lasting and durable repairs because the long-term properties of the substrate concrete and repair material can be significantly different from the properties measured at early ages. The strengthened members are frequently located in harmful media and severe environments in the service life stage, causing early degradation and performance deterioration [31]. The durability issue under environmental conditions considering monolithic samples has been observed via experimental investigation in many previous studies [32,33,34,35,36,37]. In the technical literature, few studies have also been found that observed the durability issue on composite (repaired) samples under a freeze–thaw cycle [38,39,40], elevated temperature [41,42,43,44,45], etc. Severe degradation of the interfacial properties of the composite samples was found during long-term exposure. Therefore, the long-term performance of strengthened members under severe environmental exposure should be achieved along with the short-term bonding strength of the newly overlaid PCM layer with substrate concrete from the viewpoint of practical application of this method.

Temperature and moisture are considered to be one of the major factors affecting the durability of concrete structures. Temperature in some regions of the world, especially in the tropical zone or some parts of North America, rises up to 60 °C in summer. This temperature rise due to global warming or non-sustainable development also accelerates the further deterioration of the concrete structures [46,47]. Concrete structures are also exposed to cyclic temperature instead of constant temperature. In such environmental conditions, mismatched properties between concrete and repair material may occur, and repaired deteriorated RC structures, especially the adhesive interface, become more vulnerable. Both concrete and repair materials must be durable in severe environmental exposure. Many researchers have investigated the change in the microstructure of the concrete [48,49,50] and the mechanical performance degradation of the concrete [51,52,53] after exposure to high temperature. The mechanical behavior of concrete was degraded to different degrees after high-temperature exposure. Repair materials such as PCM are also sensitive to high temperatures. In the previous experimental study [54], a flexural strength reduction of about 91.6% was reported at 90 °C. The bond strength and the constituent properties of the materials are strongly connected [55]. Therefore, the degradation of the properties of concrete and repair materials directly influenced the bonding strength under severe environmental conditions. In the past experimental study [41,42,43], the performance of the concrete–PCM interface at a different temperature levels up to 60 °C was explored experimentally for a short-duration temperature exposure. At a temperature exposure of 60 °C and 40 °C, a significant reduction in interfacial shear strength was reported compared to 20 °C. Reduction in interfacial strength was also observed under different moisture content conditions (continuous immersion in water and wetting/drying cycles) [44,45]. A detrimental influence of elevated-temperature exposure for a short duration on flexural behavior of the PCM-strengthened RC beams, such as a reduction in flexural strength, the occurrence of debonding failure, and an increase in crack width and crack spacing, was also observed experimentally [56,57,58,59]. Additional stress is generated at the bond surface when exposed to a hot and dry environment, leading to failure of the interface at a lower load [9]. Volumetric change in the two constituents’ materials (concrete and overlay materials) with higher moisture absorption also generates additional stress at the interface and creates a condition of generating cracks [13].

Our previous research [28,29,30] confirmed that the modified PCM with silica fume forms more C-S-H with strong binding force and enhances the bonding properties of substrate concrete and the repair material due to the denser microstructure at the interface. It is expected to be utilized to enhance the durability of the interface under harsh environmental conditions that should be taken into consideration for the worldwide application of this overlaying method. Examples of harsh environmental conditions include continuous and cyclic immersion in water, exposure on the hottest day, peak summer season, seasonal temperature variation, and day–night temperature variation. The aforementioned issues should be investigated for long-term design durability. As a repair material, the modified PCM with silica fume is expected to perform better in the long run under harsh environments as silica fume can cause a chemical reaction and form more C-S-H with strong binding force [30]. Due to the extreme fineness of silica fume, it fills the void, resulting in lower porosity at the interface that can hold less moisture at the beginning. Thus, it may reduce the effect of volumetric change under severe environmental conditions.

Although different individual and coupling environmental conditions have an impact on the concrete–PCM interfacial bond, the influence of some of the individual environmental conditions on the concrete–PCM interface was considered as an experimental parameter on a priority basis. Considering the aforementioned issue, Series-I of this study investigates the durability enhancement effect of silica fume of the concrete–PCM interface, considering an individual action of elevated temperature (e.g., 60 °C) for constant [short (at 60 °C for 3 days) and moderate duration (at 60 °C for 36 days)] and cyclic conditions [day–night and seasonal variation] in the laboratory. In Series-II, the performance evaluation of the bond strength considering durability enhancement was explored under different moisture content conditions [continuous immersion in water up to 96 days and up to 24 W/D cycles]. Current experimental data can provide some theoretical foundation to researchers and structural engineers on the PCM overlaying method of deteriorated concrete structure strengthening under harsh environmental conditions. The findings of this research can help the practitioner in selecting a suitable repair overlay material and the manufacturer of PCM to produce new PCM with improved functionality to use under severe environmental conditions.

## 2. Experimental Outline

### 2.1. Materials

Substrate concrete with a target compressive strength of 40 MPa was used in this experimental work. It was fabricated by mixing commercially available normal Portland cement, coarse aggregate, fine aggregate, tap water, water reducer, and air-entraining agent. The specific properties of the different constituents of substrate concrete are mentioned in our previous study [30]. The substrate concrete was chosen with relatively higher compressive strength to achieve brittle/sudden failure mode along the concrete–PCM interface. The interfacial bond strength highly depends on the mechanical properties of the constitutive materials. The reduction in the bond behavior could be more obvious with the higher compressive strength of the substrate concrete. The mix proportion used in this study to prepare one cubic meter of substrate concrete is shown in Table 1.

The second type of material was the pre-mixed PCM containing poly acrylic ester (PAE) provided by the company. All the ingredients were mixed by the manufacturer in a specific ratio to fabricate gray-color PCM powder. At the time of the application, a water–binder ratio of 15% was used in this study as per the company’s recommendation to prepare the PCM mix. X-ray fluorescence (XRF) analysis was performed to evaluate the elemental oxides of the PCM used in this study. The major constituents observed using XRF analysis were Al_2_O_3_ (4.07%), SiO_2_ (16.39%), SO_3_ (2.57%), CaO (74.07%), and Fe_2_O_3_ (2.90%).

The third material was silica fume, which was used to prepare modified PCM for proper adhesion between concrete and PCM. Gray ultrafine silica fume with a specific surface area of 15–20 m^2^/g, a relative density of 150–700 kg/m^3^, a specific gravity of 2.2–2.3 and an average particle size of 0.15 µm is used in this study. The use of silica fume causes a need for more water, as the high surface area and small particle size of silica fume demand more water. A small amount of silica fume results in less C-S-H at the interface, whereas material stagnation is caused by using a large amount of silica fume [60]. Based on the findings of previous studies [28], a 5% silica fume of PCM mass was used in this study. Since silica fume powder is not liquid and is difficult to spread in the interface, it was used by mixing into PCM powder right before overlaying and hydrating together. Superplasticizer of 1.0% of the PCM mass was mixed in preparing modified PCM repair material to prevent the formation of silica fume lumps, whereas it was not used to prepare normal PCM repair material (without silica fume) following the outcome of trial flow test by the author and previous group research in our team [28,29,30]. The use of superplasticizer has no impact on the mechanical performance of the composites as observed in the trial test by the author. The mix proportion of repair material is shown in Table 2.

### 2.2. Specimen Preparation

Cylindrical specimens of size 100 mm × 200 mm and 50 mm × 100 mm were cast and used to measure the compressive strength of concrete and PCM, respectively. A polythene sheet was used to wrap the molds to avoid the moisture evaporation. Twenty-four hours after casting, all the formworks were demolded, and the concrete specimens were submerged in a tub of water for 28 days for proper curing. The PCM specimens were cured in water for 7 days followed by dry curing for 21 days to achieve better strength of the PCM specimens. Wet curing helps the hydration process, whereas dry conditions curing benefits the formation of polymer films [61].

**Figure 1:**
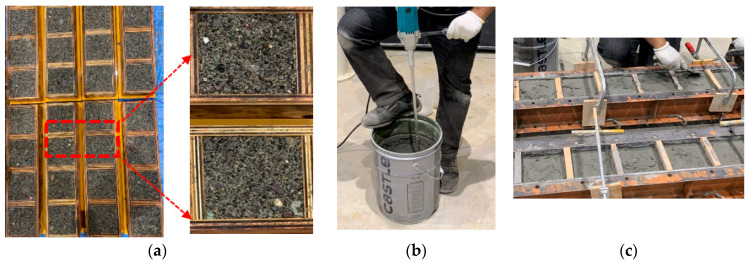
Preparation of composite specimens. (**a**) Rough concrete surface preparation. (**b**) PCM mixing using a hand mixer. (**c**) Casting of PCM in two layers.

The composite specimens were prepared by casting concrete and PCM on different dates. A portion of the concrete specimens (100 mm × 100 mm × 50 mm) was cast first, followed by pouring PCM (100 mm × 100 mm × 25 mm) to prepare the specimens of size 100 mm × 100 mm × 75 mm. Taking a large number of composite specimens into account, wooden molds were prepared for the casting of concrete of sizes 100 mm × 100 mm × 50 mm. In past research [62,63,64], the surface roughness of the concrete substrate has been extensively studied for improving the interlocking of the overlay, and a positive influence has been observed. One surface of the concrete specimens with dimensions 100 mm × 100 mm was treated to prepare a rough surface for better bonding. Following the previous studies [30,56], some amount of retarder was spread at the bottom of the molds before pouring concrete to prepare the surface roughness. It is possible to obtain a uniform rough surface with less/no damage of the substrate concrete using this method of surface roughness preparation. Before demolding, the bottom layer of the mold was removed first, and the concrete surface was exposed to the environment after 24 of casting. Due to the use of a retarder, the exposed concrete surface was not fully hard. The soft concrete surface was then sprayed with a strong jet of water until coarse aggregates were exposed to resemble a rough substrate concrete surface as shown in Figure 1a.

After demolding, the concrete was cured in wet condition for two weeks followed by dry curing for three months to resemble the existing concrete of the real infrastructure. To prepare composite specimens, cured concrete specimens of size 100 mm × 100 mm × 50 mm were put into the mold of 100 mm × 100 mm × 75 mm. The treated rough substrate concrete surface was kept upright in the mold. Good attention was paid in the following conditions before casting PCM: (i) removing of any dust with high air pressure on the treated surface and (ii) saturated concrete substrate with a dry surface for adequate bonding. Details of the procedure of the cleaning of the dust and achieving saturated surface dry condition is explained in our previous study [30]. The overlay material was mixed using a hand mixer for 1.5 min as shown in Figure 1b and troweled in two layers as shown in Figure 1c, with a time interval of 180 min to prepare composite specimens of size 100 mm × 100 mm × 75 mm. The curing methodology of the composite specimen was similar to the curing of PCM bulk cylindrical specimens previously mentioned.

### 2.3. Exposure Condition

Composite specimens were exposed in two series to study the influence of elevated temperature and moisture content in the laboratory, which resemble real environmental conditions. The specific duration of exposure under different conditions was chosen following the previous study [41,42,43,44,45] to keep the exposure duration as long as possible considering the time restraint in the laboratory and to infer longer-term applicability of the mentioned factors.

#### 2.3.1. Elevated Temperature

**Figure 2:**
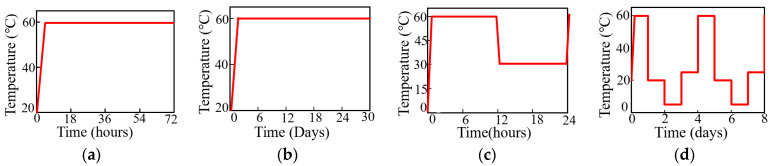
Exposure condition used to investigate the effect of elevated temperature. (**a**) T_SD_. (**b**) T_MD_. (**c**) T_DN_. (**d**) T_SV_.

In some regions of the world, the temperature fluctuates and may reach up to extreme temperature of about 60 °C from an ambient condition of 20 °C. The durability of the bonding of repair material to substrate concrete may be affected by this elevated temperature. In some regions, concrete structures are also exposed to cyclic temperature instead of constant temperature. To investigate this effect in detail, composite specimens were exposed to four different temperature variations in the laboratory as presented in Figure 2a–d.

The first condition was the exposure of composite specimens at a constant temperature of 60 °C for 3 days (Figure 2a), abbreviated as “T_SD_” (short-duration exposure). In the second condition, the composite specimen was put in an oven at a constant temperature of 60 °C for 36 days to resemble long-term exposure, abbreviated as “T_MD_” (moderate-duration exposure) (Figure 2b). These conditions were considered to simulate a specific time period in a tropical region such as summer season where temperature may rise up to 60 °C. Concrete structures were also exposed to cyclic temperature conditions instead of constant temperature conditions, which were also adopted in this study to represent real environmental conditions. Temperature change during day and night was considered by putting the composite specimen in a programmed oven at 60 °C for 12 h and at 30 °C for another 12 h, abbreviated as “T_DN_” (day and night variation) (Figure 2c). Seasonal variation of the temperature was also considered by replacing four seasons of a year with one day in the laboratory. The repaired specimens were exposed for 24 h in each condition of 60 °C in the oven, 20 °C in the water, 5 °C in the oven, and 25 °C in the oven to represent the summer season, rainy season, winter season, and at the ambient condition at 25 °C in the spring season, respectively (Figure 2d). One cycle in seasonal variation exposure was completed in 4 days. In all cases, the testing conditions were kept similar. After every exposure condition, the specimens were cooled down and tested at room temperature after cooling down. The summary of exposure conditions to study the influence of elevated-temperature exposure is shown in Table 3.

#### 2.3.2. Moisture Content

**Figure 3:**
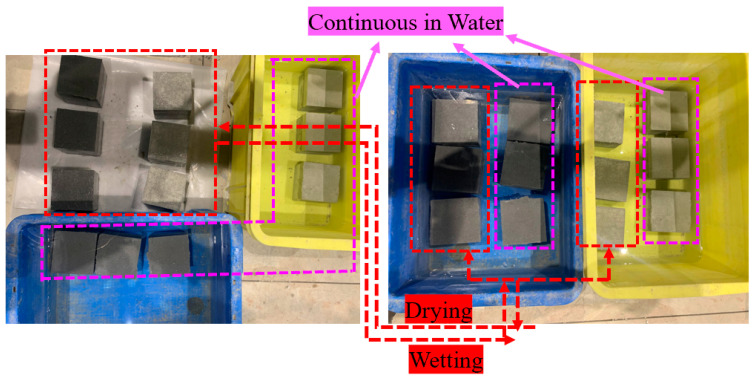
Exposure condition used for W/D cycles and continuous immersion in water.

To investigate the influence of moisture on the concrete–PCM interfacial bonding strength, composite specimens repaired with normal and modified PCM were exposed to wetting and drying cycles (W/D cycles). This condition was considered to simulate the simultaneous wet and dry exposure conditions of the RC structures in rain (wet) and summer (dry), respectively. For one W/D cycle, the specimens were submerged in water for 2 days to obtain wetting conditions followed by air exposure in the laboratory for 2 days to obtain the dry condition. Different researchers used different test methods during the wetting and drying cycles [61]. In this research work, the test was performed in a wet state after exposure to 0, 12, and 24 W/D cycles. Some RC structures experienced continuous immersion in water such as dams. Therefore, this exposure condition of continuous immersion in water at room temperature was also adopted in this study. For continuous wet conditions, the specimens were submerged in water continuously. The specimens were taken out from the water after 48 and 96 days of continuous immersion in water just before the testing, and the test was conducted in wet conditions. The process of W/D cycles and continuous immersion in water and the summary of exposure conditions and the number of specimens considered to study the influence of moisture are described in Figure 3 and Table 3, respectively.

### 2.4. Testing Procedure

The compressive strength of substrate concrete, normal PCM, and modified PCM was evaluated by conducting a compressive strength test after 28 days of curing according to the standard test method ASTM C39 [65]. The compressive strength of 41.6, 42.2, and 54.8 MPa (average of three specimens) was recorded for substrate concrete, normal PCM, and modified PCM, respectively. The modified PCM with silica fume improves compressive strength by about 30%. The compressive strength of the constituent overlay materials was also measured after 90 days of casting. An increase of 17% in strength was observed for modified silica PCM specimens due to the pozzolanic reaction of silica fume, but almost similar strength was obtained for the case of normal PCM specimens.

**Figure 4:**
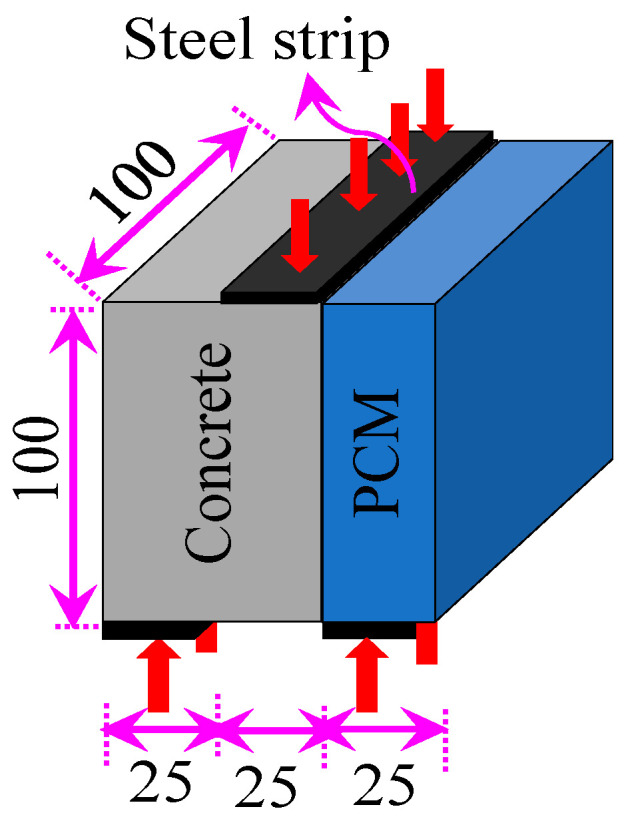
Schematic of the test method used in this research work (unit: mm).

Different bond strength tests can be found in the technical literature to access the interfacial bond, such as splitting tensile, bi-surface shear, direct single-surface shear, and slant shear [66,67,68]. Among the test methods, single or double shear and slant shear tests have been widely used to evaluate the interface shear strength. Single or double shear have the disadvantages of local compressive failure or bending crack instead of adhesive failure [69]. The slant shear test leads to higher bonding strength due to increase in friction and is greatly influenced by the size and axis at the shear plane [67]. The use of the in-house testing method of interfacial strength was not widely accepted due to the complex test setups. Momayez et al. [68] proposed a bi-surface shear strength test to evaluate interfacial shear strength that does not require any special form, can be fabricated easily, and gives less variation in results. Considering the ease of specimen preparation, simple loading method, and many specimens, the bi-surface shear test (scheme shown in Figure 4) was adopted in this research work. The interfacial shear strength of the composite specimens was evaluated by using Equation (1).

(1) $$\tau_{max}=\frac{P_{u}}{2A}$$

where $\tau_{max}$ is the interfacial shear strength, $P_{u}$ is the ultimate load, and *A* is area of the connected interface.

## 3. Test Results and Discussion

### 3.1. Influence of Elevated Temperature

#### 3.1.1. Short- and Medium-Duration Exposure

**Figure 5:**
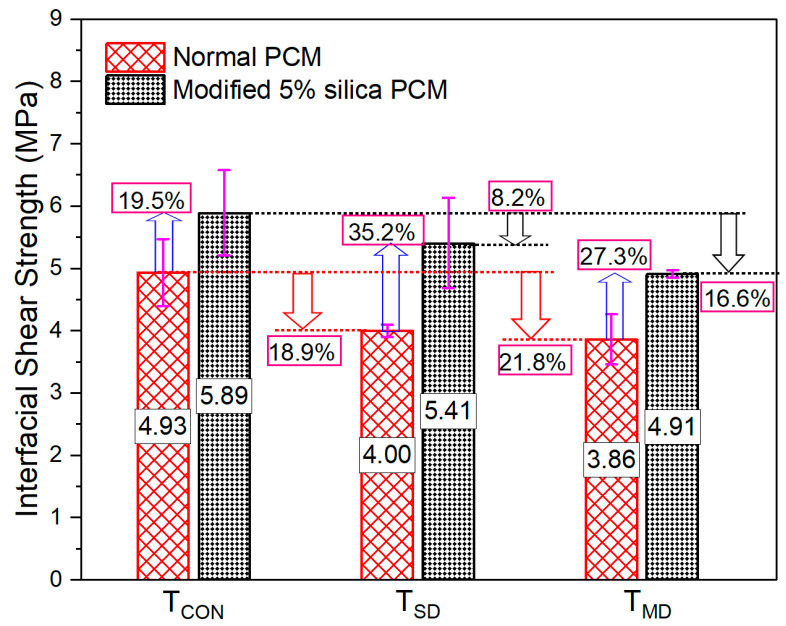
Bond strength of the composite specimens under constant elevated-temperature exposure.

The composite specimens were exposed to a constant 60 °C temperature for 3 days to study the influence of short-duration exposure (T_SD_) and for 30 days to study the influence of long time exposure (T_MD_). After the exposure, the specimens were kept in the laboratory at room temperature for 24 h to cool them down and tested at room temperature. Following the previous study [70], the specimens were cooled down before testing, in which higher bond strength reduction was observed after cooling than at elevated temperature. The maximum stress capacity of the composite specimens under T_SD_ and T_MD_ is shown in Figure 5. The reduction in interfacial shear strength with exposure to T_SD_ conditions compared to without exposure cases (T_CON_) was observed by approximately 19% and 8% for normal and modified PCM specimens, respectively. The degradation of interfacial strength further increased with the increase of exposure time (T_MD_ condition), approximately 22% and 16% for normal and modified PCM specimens, respectively. The reduction in the bond strength at elevated temperature is consistent with the results of previous studies [41,42,43,70] considering normal PCM overlay material under short- (1 day) and moderate-duration (30 days) exposures. The interface condition is one of the governing factors for the mechanical performance of the composite specimens. In a study by Xie et al. [71], the interface was stated as the most porous layer compared to the rest of the specimens. The porosity further increases and leads to a further reduction in bond strength at an elevated temperature [72]. Different thermal expansion coefficients between concrete and PCM generate high internal thermal stresses and micro-cracks, subsequently causing weak bond strength with a change in temperature [56].

The higher percentage of bond strength reduction in normal PCM specimens might be due to the presence of higher porosity at the normal PCM–concrete interface. During drying at high temperature, some of the fine pores collapsed, resulting in larger pores and reduced strength with an increase in porosity [72,73]. In all exposure conditions, higher bong strength was observed in modified PCM specimens compared to normal PCM specimens (35.3% after T_SD_ exposure and 27.3% after T_MD_ exposure), as presented in Figure 5. The formation of more hydrogen bonds with strong binding force at the interface with silica fume inclusion [30] leads to acquiring a good concrete–PCM bond, thus reducing the damage initiation under elevated temperature. In addition, a denser microstructure with silica fume incorporation improves the structure of the interface with less porosity, thus reducing the influence of elevated temperature and resulting in higher interfacial strength.

**Figure 6:**
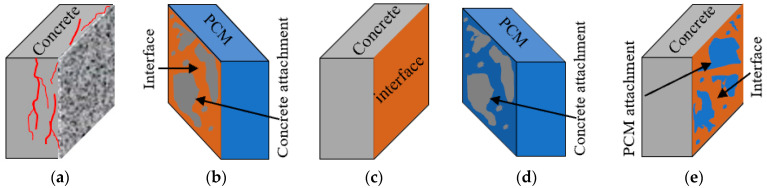
Possible fracture modes of the composite specimens. (**a**) Concrete cohesion (C). (**b**) Composite fracture (I-C). (**c**) Adhesive interface (I). (**d**) Composite fracture (C-P). (**e**) Composite fracture (I-P).

**Figure 7:**
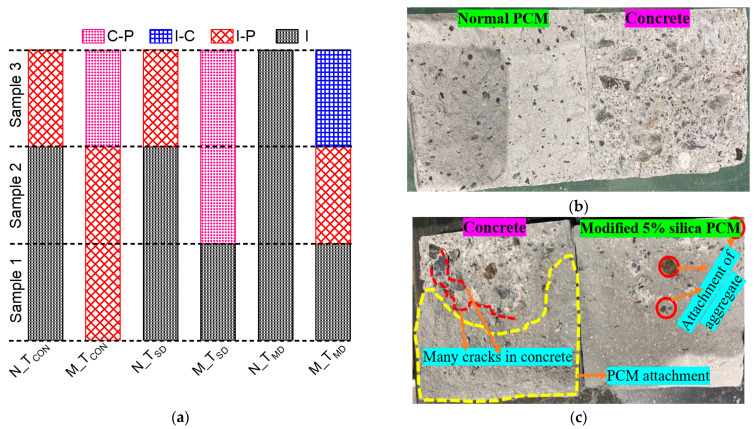
Fracture mode under constant elevated-temperature exposure. (**a**) Fracture mode. (**b**) Pure interface fracture (I) under T_MD_. (**c**) Composite fracture mode (C-P) under T_SD_.

The behavior of the composite specimens with silica fume inclusion under exposure to constant elevated temperature was also discussed in light of the fracture mode. The fracture modes are named by identifying the fracture location of the composite specimens during or after the loading test. The possible fracture modes of the composite specimens are shown in Figure 6. The fracture mode of all the composite specimens evaluated after the loading test under T_SD_ and T_MD_ along with T_CON_ is presented in Figure 7a. The specimens were designated as follows: “N” as normal PCM and “M” as PCM modified with 5% silica fume followed by the specific exposure conditions.

Adhesive pure interface (I) fracture mode was observed as a major failure type in composite specimens repaired with normal PCM, both in control (T_CON_) and exposure conditions (T_SD_ and T_MD_). Examples of adhesive pure interface (I) fracture mode are shown in Figure 7b. The composite specimens repaired with modified PCM exhibited one “(I)” fracture mode under T_SD_ and T_MD_, whereas the T_CON_ specimen do not include any “(I)” fracture mode. An example of composite fracture mode (C-P) of the composite specimens repaired with modified PCM is shown in Figure 7c. PCM attachments were found in almost the whole substrate concrete surface, which indicates the degradation of PCM more than the degradation of the adhesive interface as PCM is sensitive to high temperatures [61,74]. The higher number of specimen failures in composite fracture mode in modified PCM cases indicates higher concrete–overlay material adhesion with better durability under short- and long-duration exposure at elevated temperature.

#### 3.1.2. Day–Night and Seasonal Variation Temperature Cycle

**Figure 8:**
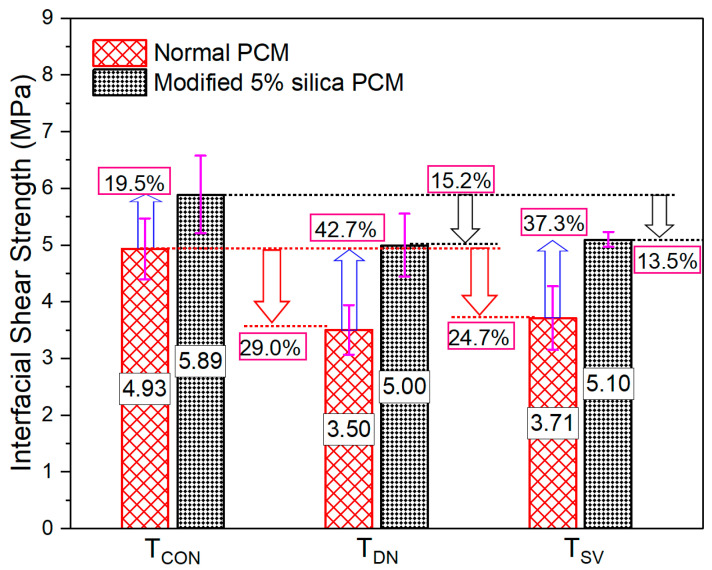
Bond strength under cyclic elevated-temperature exposure.

The real environmental condition was assumed considering cyclic temperature exposure by simulating the day–night cycle and seasonal variation for the durability of the repaired system and was provided in the laboratory in a programmed oven. The temperature was set at 60 °C for 12 h and 30 °C for another 12 h for the day–night variation case (T_DN_) and tested after 30 days of exposure. Interfacial strength reduction was observed under T_DN_ in both normal (29%) and modified PCM (15%) specimens compared to its corresponding control specimens (T_CON_), as presented in Figure 8. Similar trends of bond strength reduction of about 27% from the control specimens were also observed in the previous study [75], considering normal PCM overlay material under T_DN_ exposure for 30 days. A more detrimental influence of elevated temperature under T_DN_ (cyclic temperature cycle) were observed on bond strength than under T_SD_ or T_MD_ (constant temperature exposure) in this study and in the previous study [70,75]. Due to cyclic temperature exposure, it will cause either a contraction or residual expansion at the interface, increase strain, and subsequently reduce bond strength [73].

**Figure 9:**
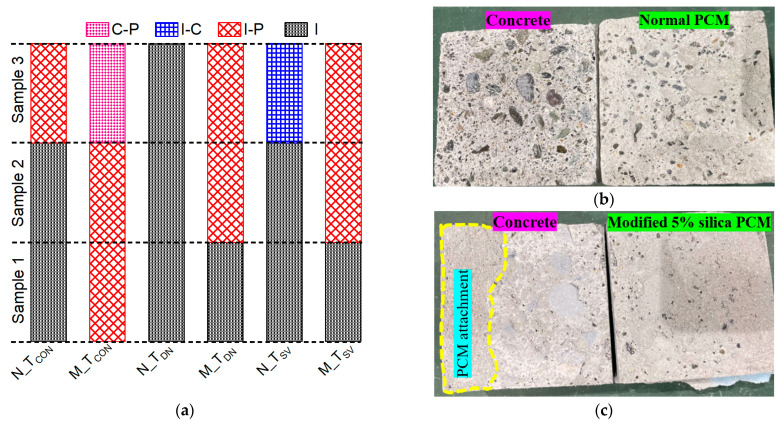
Fracture mode under cyclic temperature exposure (T_DN_ and T_SV_). (**a**) Fracture mode under T_DN_ and T_SV_. (**b**) Pure interface fracture (I) under T_DN_. (**c**) Composite mode fracture (I-P) under T_DN_.

The percentage reduction in the interfacial strength under T_DN_ compared to T_CON_ was almost half for modified PCM than that of normal PCM specimens. Strength increases of about 42% were observed with silica fume inclusion with PCM under T_DN_. This indicates durability enhancement of silica fume as a repaired mortar under elevated temperature. The fracture mode was observed as “(I)” for all normal PCM specimens under T_DN_, whereas two out of three specimens with modified PCM exhibited composite (I-P) fracture mode, as presented in Figure 9a. Even though the PCM is very sensitive to high temperatures [54,74], the occurrence of “(I)” fracture in normal PCM specimens (Figure 9b) indicates that the normal PCM–concrete interface is more vulnerable under cyclic temperature condition exposure. In contrast, in the pictural view of the fracture surface of the modified PCM specimen, the PCM attachment on the concrete side was observed as shown in Figure 9c, which verifies the degradation of PCM more than the adhesive interface at elevated temperature.

The temperature variation of four seasons in a year (summer, rainy, winter, and spring) was simulated by exposing the specimen to each environment for 24 h in the laboratory. The mechanical loading test was performed to evaluate the bonding strength after 12 cycles of exposure (48 days, 4 days to complete one cycle). The strength reductions of 24.7% and 13.5% were observed under T_SV_ for normal and modified PCM, respectively, compared to T_CON_ specimens, as presented in Figure 8. Strength reduction of about 24% was observed in the previous study [75] after the 10th cycle of seasonal variation exposure considering normal PCM overlay material. Higher bond strength reduction was observed in the T_SV_ condition than in T_SD_ or T_MD_. Similar trends of strength reduction under cyclic and constant temperature exposure were also observed in the previous study [70,75]. This might be due to the fact that the degraded polymers in the PCM cannot recover fully due to the cyclic temperature variation [74]. Owing to cyclic conditions, additional stress may generate at the interface and create a condition of generating micro-cracks, resulting in a reduction in the bond strength. Though the exposure duration under T_SV_ is higher than T_DN,_ the later exposure condition resulted in more degradation of interfacial strength. This might be due to the possibility that during T_SV_ exposure, the deteriorated PCM at elevated temperature may restructure itself after cooling [76], resulting in less detrimental influence of seasonal variation than day–night variation exposure.

The outcome of higher interfacial strength (about 37%), as presented in Figure 8, and a smaller number of “(I)” fracture modes, as presented in Figure 9a, under T_SV_ in modified PCM specimens suggested a positive influence of durability enhancement of silica fume with PCM as a repaired mortar. This might be attributed to the formation of more hydrogen bonds at the interface with a strong binding force, as observed in our previous study [30] and by generating a denser microstructure of the interface (micro-filler effect) due to the incorporation of extremely fine and reactive silica fume with PCM. Conclusively, silica fume improves the durability of the composite specimen under cyclic temperature conditions.

### 3.2. Influence of Moisture Content

#### 3.2.1. Wetting and Drying Cycles

**Figure 10:**
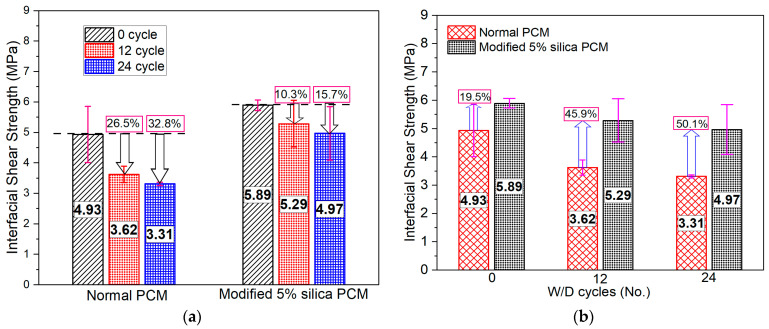
Bonding strength under moisture content (W/D cycles). (**a**) Degradation of interfacial strength. (**b**) Influence of silica fume inclusion.

The composite specimens were exposed to a cycle of 2 days of wetting followed by 2 days of drying conditions to study the influence of simultaneous wetting and drying (W/D) conditions on the interfacial strength. One W/D cycle was completed in four days and the mechanical loading test was performed in wet conditions to evaluate the bonding strength after 0, 12, and 24 cycles of exposure. The interfacial strength was reduced further with the increase in the W/D cycles both in normal and modified PCM specimens, as presented in Figure 10a. About 11% strength reduction after the first W/D cycle and about 15% strength reduction after 12th W/D cycle considering normal PCM overlay material were observed in the previous study [75], indicating similar trends of strength reduction as observed in this study. As the interface is the most porous layer compared to the rest of the specimens, a higher degree of saturation at the interface degrades the mechanical properties and leads to a reduction in the bond strength [77]. The intensified pore water exerts pressure and generates micro-cracks during the external force application, subsequently degrading the strength. Absorbed water within the finest pores of cement gel also generates stress and lowers the energy barrier for bond rupture [73].

In the W/D cycle condition, moisture at the interface exerts water pressure, causing micro-cracks that deteriorate the interfacial bonding during continuous wetting and drying, resulting in a significant reduction in the bonding strength. The strength reduction was only about 15% in the modified PCM specimen, whereas it was more than 30% in the normal PCM specimen compared to its corresponding control specimens tested before any exposure condition (0 cycle). This is due to the higher level of moisture in the normal PCM–concrete interface that exerts pressure from inside during loading and increases the level of damage. To evaluate quantitatively the durability enhancement effect of silica fume, the bond strength with/without silica fume was compared under different W/D cycle exposure and presented in Figure 10b. In all exposure states of W/D cycles, the modified PCM specimens showed higher interfacial strength than normal PCM specimens. The use of silica fume reduces the porosity of the interface due to its extreme fineness, thus reducing the moisture presence in the void space. This will cause less presence of moisture in the void space and reduce the impact of water pressure during continuous W/D, thus decreasing the damage degradation under W/D cycles. In addition, the formation of more C-S-H with strong binding force with silica fume inclusion helps to acquire an adequate bond between the concrete substrate and repair material, thus reducing the damage initiation and propagation along the interface under W/D cycles.

**Figure 11:**
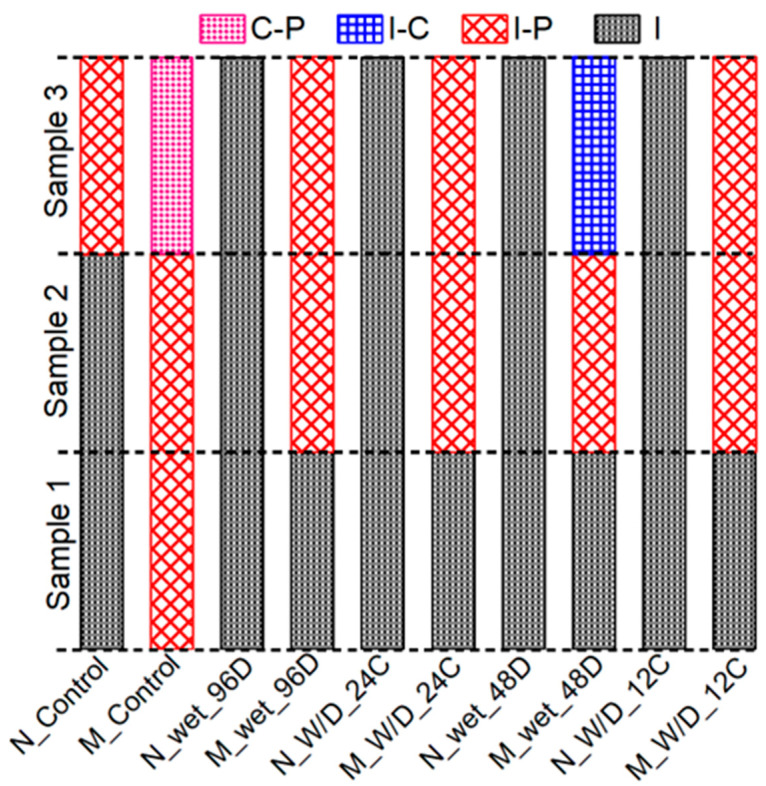
Fracture mode under influence of moisture.

**Figure 12:**
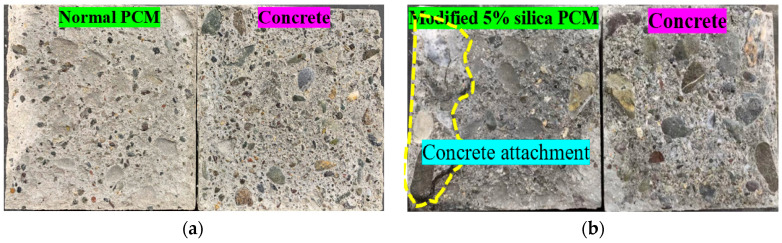
Sample fracture mode of the composite specimen under W/D cycle exposure. (**a**) Pure interface fracture (I) mode. (**b**) Composite fracture (I-C) mode.

The fracture mode under the influence of moisture is presented in Figure 11. The acronym designation adopted as follows: “N” or “M” refers to normal or modified PCM followed by exposure condition and exposure duration. For example, N_W/D_12C refers to normal PCM specimens exposed under wetting and drying conditions and tested after 12 cycles of exposure. Interface fracture (I) was the dominant fracture mode for normal PCM specimens irrespective of the number of W/D cycles, whereas the specimen with modified PCM exhibited only one “(I)” fracture and two composite, (I-P) or (I-C), fracture modes. As an example, the pictural view of the fracture surface after the loading test of the normal PCM [pure interface fracture (I)] and modified PCM specimen [composite mode fracture (I-C)] is shown in Figure 12a,b, respectively. A higher number of “(I)” fracture modes in normal PCM specimens than in modified PCM specimens indicates higher modified PCM overlay–concrete adhesion with better durability.

#### 3.2.2. Continuous Immersion in Water

**Figure 13:**
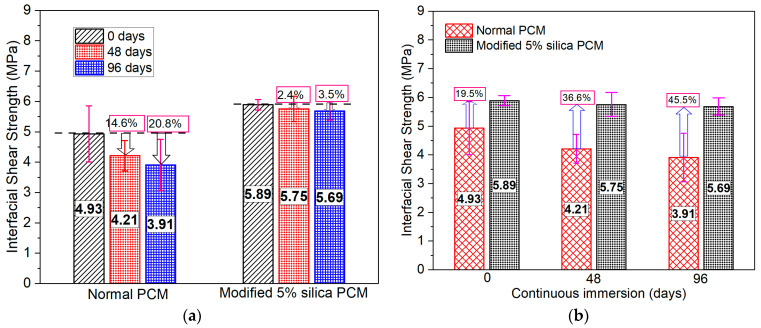
Bonding strength under moisture (continuous wetting). (**a**) Degradation of interfacial strength. (**b**) Influence of silica fume inclusion.

The composite specimens were immersed in water to study the influence of continuous immersion in water on the interfacial strength. The specimens were taken out of the curing tank after 48 and 96 days of exposure and the test was conducted immediately in wet conditions. The interfacial strength reduction of about 20% was observed after 96 days of exposure compared to control cases tested before any exposure condition (zero immersion day) of normal PCM specimens, whereas its effects were found marginal in modified PCM specimens, as presented in Figure 13a. The interfacial strength was within 6% of the strength of control specimens in the previous study [75] after 48 days of continuous immersion in water. Similar observations were also found in this study up to 48 days of exposure in normal PCM overlay material.

Extra moisture at the interface of normal PCM specimens due to higher porosity loosens the cohesion of the polymers by swelling and dissolving the polymer films, resulting in strength reduction [78]. This resulted in quite limited absorbed water at the modified PCM–concrete interface during continuous wetting due to the lowering of porosity with silica fume inclusion; thus, its effect on modified specimens is marginal. The durability enhancement effect of silica fume was evaluated after 0, 48, and 96 days of continuous wetting of with/without silica fume specimens, as presented in Figure 13b. The higher interfacial bonding strength in modified PCM than normal PCM specimens at all exposure states indicates that the exposure condition of continuous wetting has very little effect on the modified PCM–concrete interface than the normal PCM–concrete interface, which has a significant effect.

**Figure 14:**
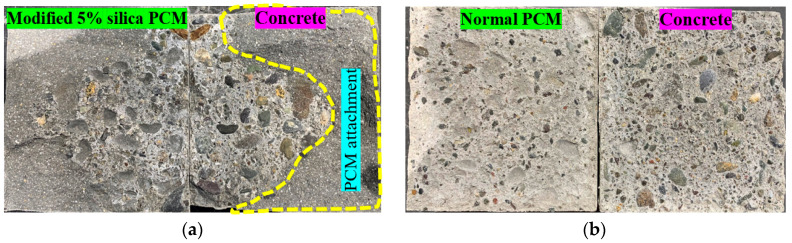
Sample fracture mode of the composite specimen under continuous wetting conditions. (**a**) Composite fracture (I-P) mode. (**b**) Pure interface fracture (I) mode.

The fracture mode of all the specimens tested under the exposure of continuous immersion is shown in Figure 11. As an example, the pictural view of the fracture surface after the loading test of the normal and modified PCM specimen is presented in Figure 14a,b, respectively. The number of “(I)” fracture modes in modified PCM specimens was less than in normal PCM specimens, which indicates higher modified PCM overlay–concrete adhesion. This might be due to the fact that the environment for the proper hydration and curing improved for the silica fume modified PCM with the continuous immersion in water. In addition, free alkali after the hydration of cement reacts with the silica compound to form an additional C-S-H hydrate under the water-supplied condition, resulting in better bonding performance of the composite under continuous wetting conditions. Conclusively, the modified PCM is very important to achieve a good interfacial bond with concrete under continuous wetting.

**Figure 15:**
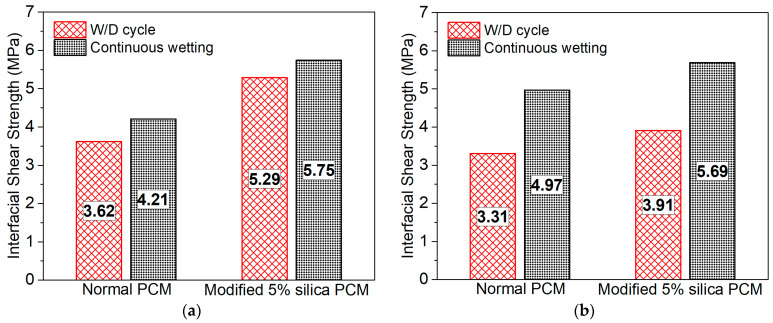
Influence of exposure types under moisture content on interfacial bond strength: (**a**) 48 days of exposure and (**b**) 96 days of exposure.

The influence of moisture content after the same length of exposure under continuous immersion in water was observed to be lower than the W/D cycle conditions, as shown in Figure 15a,b. The hydration and curing environment improved for PAE PCM with the continuous immersion in water. In addition, in continuous immersion cases, the hydroxyl group in the concrete reacts to form an additional hydrogen bond with the PCM under continuous water supplied conditions [79]. Therefore, it gives extra overlay material–concrete adhesion and improved composite action of the repaired specimens.

### 3.3. Statistical Analysis

Statistical analyses of the experimental data of interfacial strength obtained from the loading test were performed using a one-way ANOVA. Significant criteria were imposed considering the *F-value* to evaluate the durability enhancement effect of silica fume PCM as a repair mortar under the influence of influencing factors (elevated temperature and moisture content) on the interface of both normal and modified PCM specimens. The details of the calculation process/formulas of the parameters of the ANOVA table, as shown in Table 4, are presented in the literature [80].

The degree of influence of the considered factor (elevated temperature and moisture under different head) were evaluated by comparing the calculated *F-value* using the ANOVA table and the critical *F-value* (*F_x_*) obtained using *F* distribution at 1% (*F*_0.01_), 5% (*F*_0.05_), and 10% (*F*_0.1_) significance levels. The degree of influence of the considered factor can also be obtained from the *p*-value at different percentages of significance level. The significance criteria used in this research work are presented in Table 5.

The statistical analyses result of the experimental data using a one-way ANOVA is presented in Table 6 and Table 7 for the moisture content and elevated temperature, respectively. The critical *F-value* (*F_x_*) was evaluated as 3.46, 5.14, and 10.92 using *F* distribution at 1% (*F*_0.01_), 5% (*F*_0.05_), and 10% (*F*_0.1_) significance levels, respectively. The calculated *F-value* for the normal PCM specimens under W/D cycle was 14.92, which was higher than the critical *F-value* [*F*_0.1_ (10.92)], indicating a highly significant effect of the exposure condition on the interfacial bonding strength. Similarly, the level of significance of the other exposure condition under moisture were obtained by comparing the calculated and critical *F-value* and tabulated in Table 6. The calculated *F-value* for the modified PCM specimens (5.17) under W/D cycle exposure was smaller than the normal PCM specimens (14.92). This confirmed that the exposure condition of W/D cycle has less effect on the modified PCM–concrete interface compared to the interface of normal PCM–concrete that have a highly significant effect under W/D cycle. The statistical analyses result also confirmed that the exposure condition of continuous wetting (Table 6) and elevated temperature (both constant and cyclic condition) (Table 7) has very little effect on the modified PCM–concrete interface compared to the interface of normal PCM–concrete, which has a significant effect. Conclusively, the statistical analysis results along with experimental data imply the positive influence of modified PCM as a repair material to achieve good interfacial bonding strength under both W/D cycle and continuous immersion in water.

## 4. Conclusions

The influence of elevated temperature fluctuation and moisture content on the PCM interfacial bonding strength with/without silica fume was investigated. Four temperature exposure conditions with a maximum temperature level of 60 °C to simulate short-duration, moderate-duration and cyclic (day–night and seasonal variation) conditions and two moisture content exposure conditions to simulate continuous immersion in water and simultaneous wetting and drying (W/D) cycle up to 96 days were investigated and the following conclusions can be drawn.

- The concrete–PCM interfacial bond strength significantly reduces with the exposure at elevated temperature (60 °C).
- The use of normal PCM (without silica fume) alone as an overlay material results in a higher percentage decrease in bond strength under both short-duration (3 days) and long-duration (30 days) exposure at constant 60 °C temperature. The combined use of PCM and silica fume significantly decreases the reduction percentage of bond strength. A delay in the occurrence of interface fracture (at higher load) and the higher number of specimen failures in composite fracture mode in modified PCM cases indicate higher concrete–overlay material adhesion with better durability under constant temperature exposure.
- The reduction percentage further increases under day–night and seasonal variation exposure conditions, resulting in more detrimental influence of the cyclic elevated temperature on the interfacial bonding strength. The use of silica fume with PCM led to an optimal solution in terms of bond strength, PCM–concrete adhesion, thus improving the durability of the concrete–PCM interface under cyclic temperature conditions. Both W/D cycle and continuous immersion conditions have a significant effect on the bond properties of the concrete-to-normal PCM interface. Compared to normal PCM mortar, however, use of modified silica PCM mortar significantly improves bond strength and led to more ductile failure due to the multiple cracking behavior, which dispersed more uniformly the shear stress transfer at the concrete–PCM interface under moisture content exposure.
- The influence of moisture content after the same length of exposure under continuous immersion results in lower a reduction in PCM interfacial strength with and without silica fume compared to the exposure under wetting and drying cycle.

The concrete–PCM interface is the most critical component in the PCM overlaying method. To evaluate the overall performance of the strengthened structures, it is necessary to consider the effects of environmental degradation on the interface. The obtained data confirm the suitability of the PCM overlaying method with silica fume under harsh environments as it provides adequate concrete–overlay material bond and enhances adhesion and durability under elevated temperature and moisture. However, the results from this study are restrained to a limited duration of exposure. Additional data should be developed with a longer duration of exposure of about 2–3 years in the future to infer longer-term applicability and to predict the service life of strengthened structures accurately under harsh environmental conditions.

## Figures and Tables

**Table 1:** Mix proportion of one cubic meter of substrate concrete.

W/C (%)	Amount (kg/m^3^)					Air-Entraining Agent (mL/m^3^)	Compressive Strength (MPa)
	Water	Cement	Fine Aggregate	Coarse Aggregate	Water Reducer		
40	160	400	818	1018	8	187.5	41.6

**Table 2:** Mix proportion of repair material.

Overlay Material	Water/PCM (%)	Silica Fume (% of PCM Mass)	Superplasticizer (% of PCM Mass)
Normal PCM	15.0	0	0
Modified 5% silica PCM	15.0	5	1

**Table 3:** Summary of exposure conditions and the number of specimens.

Specimen Level				Overlay Constituents	
				Normal PCM	Modified 5% Silica PCM
**Exposure conditions**	Elevated temperature (Series-I)	0 day	T_Con_	3	3
		3 days	T_SD_	3	3
		36 days	T_MD_	3	3
		30 cycle	T_DN_	3	3
		12 cycle	T_SV_	3	3
	Moisture content (Series-II)	0 cycle	W/D cycle	3	3
		12 cycle		3	3
		24 cycle		3	3
		0 day	Continuous immersion	3	3
		48 days		3	3
		96 days		3	3

**Table 4:** Calculation procedure of one-way ANOVA.

Source of Variation	Sum of Squares Deviation (*SS*)	Degree of Freedom (*DF*)	Mean Square Deviations (*MS*)	*F-Value*
Between groups	S_A_	r-1	MS_A_ = S_A_/(r-1)	MS_A_/MS_E_
Within groups	S_E_	n-r	MS_E_ = S_E_/(n-r)	
Total variance	S_T_	n-1		

Note: “r” refers to levers number of influencing factor, and “n” refers to the total outcomes of different levers for influencing factor.

**Table 5:** Significant criteria of each influencing factors.

Criteria	Result
If *F* > *F*_0.01_ (r-1,n-r)	Highly significant effect
If *F*_0.01_ (r-1,n-r) > *F* > *F*_0.05_ (r-1,n-r)	Significant effect
If *F*_0.05_ (r-1,n-r) > *F* > *F*_0.1_ (r-1,n-r)	Little effect
If *F* < *F*_0.10_ (r-1,n-r)	No or very little effect

**Table 6:** Statistical analysis to evaluate the influence of moisture content under different heads.

Factors and Exposure Conditions			Interfacial Shear Strength (MPa)			*SS*	*DF*	*MS*	*F-Value*	*F_x_*	Level of Significance
W/D cycles	NPCM	0 cycle	5.60	4.27	6.05	5.98	1	5.98	14.92	*F*_0.1_ = 3.46 *F*_0.05_ = 5.14 *F*_0.01_ = 10.92	Highly significant
		12 cycle	3.90	3.36	3.61	2.80	7	0.40			
		24 cycle	3.28	3.37	3.27	--	--	--			
	SPCM	0 cycle	6.27	6.30	5.11	2.70	1	2.70	5.17		Significant
		12 cycle	4.52	6.05	5.29	3.66	7	0.52			
		24 cycle	5.47	4.46	3.72	--	--	--			
Continuous immersion	NPCM	0 day	5.60	4.27	6.05	5.20	1	5.20	9.92		Significant
		48 days	3.93	3.92	4.78	3.67	7	0.53			
		96 days	4.14	3.67	2.51	--	--	--			
	SPCM	0 day	6.27	6.30	5.11	0.07	1	0.07	0.31		No or very little
		48 days	5.41	6.22	5.63	1.46	7	0.21			
		96 days	5.48	5.54	6.03	--	--	--			

Note: “NPCM” denotes normal PCM specimens, and “SPCM” denotes modified 5% silica PCM specimens.

**Table 7:** Statistical analysis to evaluate the influence of elevated temperature under different heads.

Factors and Exposure Conditions			Interfacial Shear Strength (MPa)			*SS*	*DF*	*MS*	*F-Value*	*F_x_*	Level of Significance
Constant temperature Exposure	NPCM	T_CON_	5.60	4.27	6.05	3.14	1	3.14	8.07	*F*_0.1_ = 3.46 *F*_0.05_ = 5.14 *F*_0.01_ = 10.92	Significant
		T_SD_	6.51	3.93	4.07	2.72	7	0.39			
		T_MD_	3.83	3.47	4.28	--	--	--			
	SPCM	T_CON_	6.27	6.30	5.11	1.44	1	1.44	3.62		No or very little
		T_SD_	6.04	5.57	4.62	2.80	7	0.40			
		T_MD_	4.50	4.58	5.65	--	--	--			
Cyclic temperature	NPCM	T_CON_	5.60	4.27	6.05	3.80	1	3.80	5.59		Significant
		T_DN_	3.42	3.97	3.11	4.75	7	0.68			
		T_SV_	4.08	3.99	3.07	--	--	--			
	SPCM	T_CON_	6.27	6.30	5.11	0.95	1	0.95	0.31		No or very little
		T_DN_	4.60	5.39	2.50	1.77	7	0.25			
		T_SV_	5.03	5.25	5.01	--	--	--			

## Data Availability

All data generated or used during the study appear in the submitted article.
